# Comparative evaluation of hybrid and individual models for predicting soybean yellow mosaic virus incidence

**DOI:** 10.1038/s41598-025-99427-5

**Published:** 2025-05-06

**Authors:** Yunish Khan, Vinod Kumar, Amel Gacem, Anurag Satpathi, Parul Setiya, Kumari Surbhi, Ajeet Singh Nain, Dinesh Kumar Vishwakarma, Ahmad J. Obaidullah, Krishna Kumar Yadav, Ozgur Kisi

**Affiliations:** 1 Department of Mathematics, Statistics and Computer Science, College of Basic Science and Humanities, G.B. Pant University of Agriculture and Technology, Pantnagar, Uttarakhand 263145 India; 2 Department of Physics, Faculty of Sciences, University 20 Août 1955, Skikda, Algeria; 3 Department of Agrometeorology, College of Agriculture, G.B. Pant University of Agriculture and Technology, Pantnagar, Uttarakhand 263145 India; 4 Department of Plant Pathology, G.B. Pant University of Agriculture and Technology, Pantnagar, Uttarakhand 263145 India; 5 Department of Irrigation and Drainage Engineering, G.B. Pant University of Agriculture and Technology, Pantnagar, Uttarakhand 263145 India; 6 Department of Pharmaceutical Chemistry, College of Pharmacy, King Saud University, P.O. Box 2457, Riyadh, 11451 Saudi Arabia; 7 Department of VLSI Microelectronics, Saveetha School of Engineering, Saveetha Institute of Medical and Technical Sciences (SIMATS), Saveetha University, Chennai, Tamil Nadu 602105 India; 8 Environmental and Atmospheric Sciences Research Group, Scientific Research Center, Al-Ayen University, Thi-Qar, Nasiriyah, 64001 Iraq; 9 Department of Civil Engineering, Technical University of Applied Sciences Lübeck, 23562 Lübeck, Germany; 10 Department of Civil Engineering, Ilia State University, Tbilisi, 0162 Georgia; 11 School of Civil, Environmental and Architectural Engineering, Korea University, Seoul, 02841 South Korea; 12 Division of Agrometeorology, Sher-e-Kashmir University of Agricultural Sciences and Technology of Kashmir, Shalimar, Srinagar, Jammu and Kashmir 190025, India

**Keywords:** Soybean yellow mosaic virus, SMLR, ANN, LASSO, RR, Elastic net (ELNET), Principal component analysis (PCA), Mathematics and computing, Plant sciences

## Abstract

Forecasting the severity of crop diseases is crucial for agricultural productivity and can be achieved through statistical and machine learning techniques. Predictive models that consider weather conditions during critical growth stages of crops have shown promising accuracy. However, selecting the most suitable forecasting model remains a challenge. This research investigates the impact of various weather factors on Soybean Yellow Mosaic Virus (SYMV) incidence. Specifically, six multivariate models Stepwise Multiple Linear Regression (SMLR), Artificial Neural Networks (ANN), Least Absolute Shrinkage and Selection Operator (LASSO), Ridge Regression (RR), Elastic Net (ELNET), and SMLR_ANN both direct and with Principal Component Analysis (PCA)-were developed using 20 years of data (2001 to 2020) to predict the severity of soybean disease in Pantnagar, Uttarakhand. The dataset was divided into two parts, with 80% used for calibration and the remaining 20% for validation. Model accuracy was evaluated using several statistical criteria, including R², RMSE, nRMSE, MAE, PE, and EF. The results indicated that the PCA-SMLR-ANN (nRMSE_val_ = 0.76%) model was the most effective predictor of soybean disease severity, closely followed by the PCA-ANN (nRMSE_val_ = 3.67%) model. Hybrid models such as PCA-SMLR-ANN and PCA-ANN outperformed individual models like SMLR (nRMSE_val_ = 47.72%) and ANN (nRMSE_val_ = 6.82%). The performance ranking of the models is as follows: PCA-SMLR-ANN ≈ PCA-ANN ≈ SMLR-ANN ≈ ANN > PCA-ELNET > PCA-Ridge > ELNET ≈ RR > PCA-LASSO > LASSO > PCA-SMLR ≈ SMLR. These findings highlight the superior efficiency of hybrid models in predicting soybean disease severity based on weather indices in the study region.

**Supplementary Information:**

The online version contains supplementary material available at 10.1038/s41598-025-99427-5.

## Introduction

Soybean (*Glycine max* L. Merrill) is one of the most important oilseed crops worldwide. Brazil is the largest producer, contributing 38% of global production, followed by the United States at 31%, while India ranks fifth among the leading soybean-producing nations^1^. In the hilly regions of the Northwestern Himalayas, particularly in Uttarakhand, soybean is a primary *Kharif* crop, with the state accounting for approximately 90–95% of the total soybean acreage and production^2^. However, soybeans are susceptible to more than 300 diseases that can significantly affect their growth and yield^3,4^. Among these, Soybean Yellow Mosaic Virus (SYMV) is a highly destructive viral disease that threatens soybean production in India and neighbouring countries, including Sri Lanka, Bangladesh, Pakistan, and Thailand^5^.

SYMV is a severe disease affecting soybean crops, leading to substantial economic losses by reducing crop productivity. The disease is transmitted by whiteflies (*Bemisia tabaci*), whose population dynamics are heavily influenced by weather conditions such as temperature, relative humidity, rainfall, and sunshine hours^6^. Studies indicate that high rainfall in July, followed by dry conditions in August, combined with specific temperature and humidity ranges, favour the development and spread of the disease^7–9^. The anticipated timeframe for disease manifestation, the primary weather factors influencing its spread, and the combined effects of weather and disease on yield are crucial for effective disease management. Climate change further complicates disease prediction, as rising global temperatures are expected to increase pest-related agricultural losses by 10–25% per degree of warming^10^.

To mitigate the impact of SYMV, accurate disease forecasting models are essential for timely interventions and disease control strategies^11,12^. Traditional statistical models, such as Multivariate Linear Regression (MLR) have been used to predict crop disease severity^13–16^. However, they often struggle to capture the complex interactions between weather variables and disease incidence. Advanced machine learning (ML) techniques, including Support Vector Machines (SVMs), Artificial Neural Networks (ANNs), deep learning models, and Convolutional Neural Networks (CNNs), have demonstrated superior prediction accuracy compared to traditional methods^17–23^. Moreover, Bayesian theory, fuzzy logic, and ANN-based models have been widely applied for crop pest and disease prediction^24–26^.

Recent research highlights the importance of hybrid models, which integrate multiple modeling approaches, in achieving improved predictive performance^27–33^. Hybrid models combining statistical techniques and neural networks have shown enhanced accuracy in time-series forecasting, making them a promising alternative for predicting SYMV spread in Udham Singh Nagar^34^. Additionally, phenological weather indices, which track key crop growth stages regarding environmental conditions, have proven valuable tools in forecasting crop disease severity^35–39^. Despite advancements in predictive modeling, challenges remain in selecting the most suitable model for disease forecasting. Multiple interacting factors influence disease spread; individual models often fail to capture these complex relationships^40–43^. While machine learning and deep learning models offer promise, studies comparing their effectiveness have shown mixed results. For example, Gill et al.^44^ found that machine learning models like XGBoost and Random Forest outperformed deep learning models in predicting soybean traits, emphasizing careful model selection. Furthermore, Sandra et al.^45^ demonstrated that genetic variations among SYMV strains influence disease spread, adding another layer of complexity to predictive modeling.

Previous studies have compared the impact of weather indices on disease severity, but limited research has focused on the comparative performance of hybrid models versus individual models in forecasting SYMV outbreaks^40^. This study addresses this gap by developing and evaluating multiple hybrid and individual models to predict the severity of SYMV in Udham Singh Nagar, Uttarakhand. The models examined include:

- Individual models: Stepwise Multiple Linear Regression (SMLR), ANN, Least Absolute Shrinkage and Selection Operator (LASSO), Ridge Regression, and Elastic Net.
- Hybrid models: PCA-SMLR, PCA-ANN, PCA-LASSO, PCA-RR, PCA-ELNET, SMLR-ANN, and PCA-SMLR-ANN.

The objective is to identify the most effective model for disease forecasting, providing valuable insights for improved disease management and crop protection strategies in soybean cultivation. The outcomes of this study will contribute to advancing agricultural disease prediction methodologies, helping farmers and policymakers implement more precise and timely disease management interventions.

## Materials and methods

### Study area

**Fig. 1:**
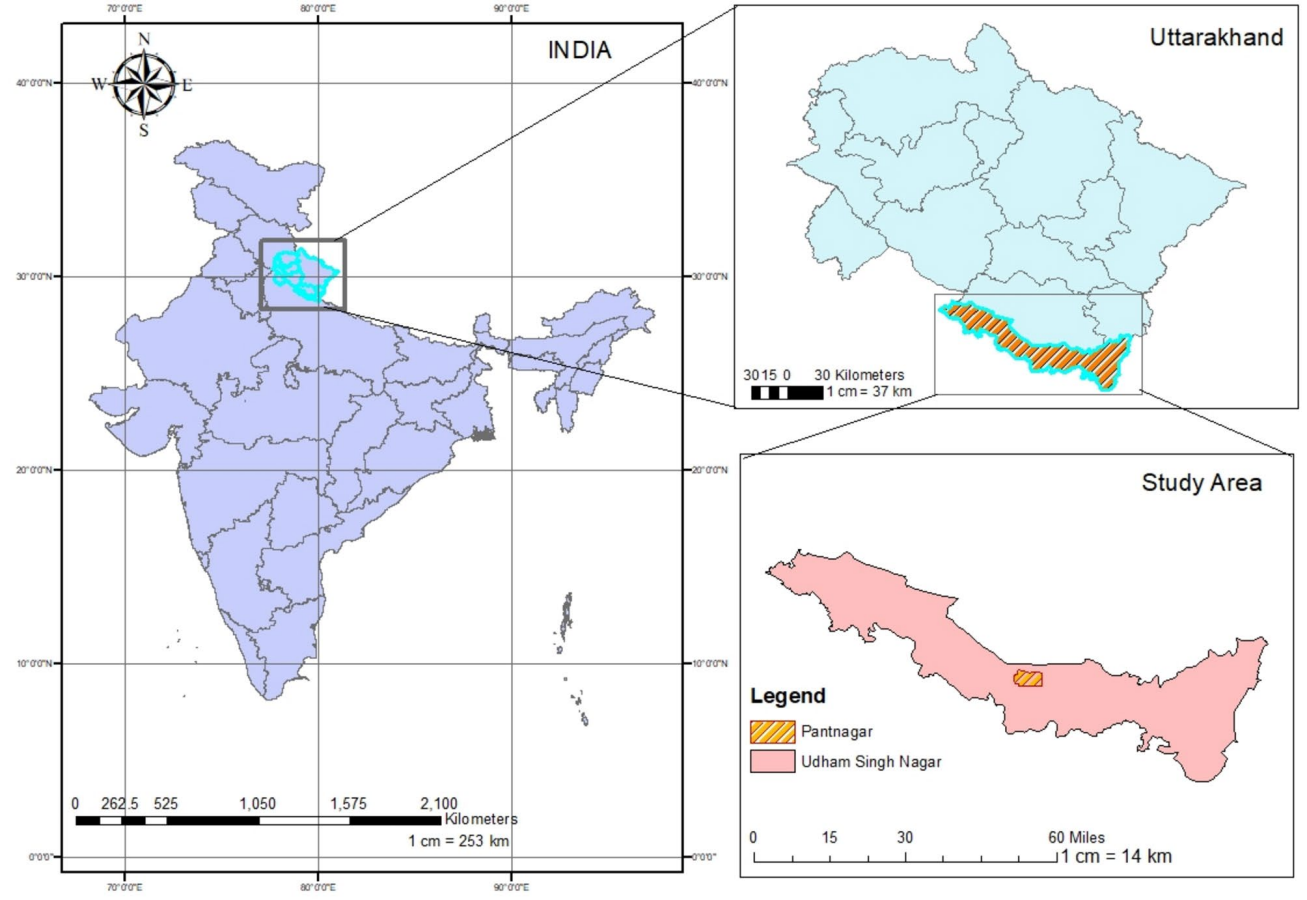
Location map of study area.

Models for predicting soybean disease severity are designed using data on disease severity in kharif soybeans and the weather data of GBPUAT, Pantnagar, Uttarakhand. The Pantnagar region lies in the Tarai belt of Uttarakhand state of India at 29° 3’ N latitude and 79° 31’ E longitude at 243 m above the mean sea level (Fig. 1). This region benefits from fertile soil and adequate moisture retention, contributing to its agricultural productivity.

### Data collection

Time series data of soybean disease severity of 20 years (2001–2020) were obtained from the Epidemiology and Molecular Diagnostics Laboratory, Department of Plant Pathology, GBPUAT, Pantnagar, Uttarakhand, India. The weather variables related data were gathered from the Meteorological Observatory, Department of Agro-meteorology, GBPUAT Pantnagar, Uttarakhand, India. Soybean cultivar “PS-1092” was selected for this study. In India, soybean is grown during the kharif season (June to November), and disease appears with the availability of favorable weather conditions.

### Development of weighted and unweighted weather indices

For this study, out of the complete dataset covering 20 years, 16 years of data were used to train the models, while the remaining 4 years’ data were reserved for testing the models^46,47^. Average values were derived from the daily weather data and these averages were subsequently employed to calculate both weighted and unweighted weather indices^48^. Two weather indices were created for each weather variable: unweighted and weighted. Unweighted indices were obtained by adding up individual weather variables or their combinations. In contrast, weighted indices were calculated by multiplying the individual or combined weather variables by their correlation with the dependent variable and then summing the results. To generate these indices, each of the nine weather variables was paired with the others, forming 45 unique combinations. Each combination was then computed separately for both weighted and unweighted indices, resulting in 90 weather indices (45 unweighted and 45 weighted). Using derived weather indices instead of raw weather variables is justified by their ability to enhance predictive performance. The unweighted indices directly represent raw weather variables, ensuring their contribution remains intact. However, incorporating weighted indices significantly improves model accuracy by integrating correlation-based weightage. This method ensures that indices with stronger positive or negative correlations contribute more to the model predictions. In comparison, those with weaker correlations naturally have lower influence due to their lower weightage. The formulas utilized by Khan et al.^38^ and Satpathi et al.^49^ to compute the weighted and unweighted weather indices are denoted by Eqs. (1) and (2), respectively. Weather indices for l^th^ year are given by:

Unweighted weather indices:

1 $$\:{W}_{ij}={\sum\:}_{w=1}^{n}{X}_{iw}{Z}_{iw}$$

Weighted weather indices:

2 $$\:{W}_{ij}={\sum\:}_{w=1}^{n}{{r}^{j}}_{iw}{X}_{iw{\prime\:}}\:\:{Z}_{iw}$$

where $\:{X}_{iw}$ and $\:{Z}_{iw}$ is the value of i^th^ weather variable in the w^th^ week, $\:{{r}^{j}}_{iw}$ is the correlation coefficient between detrended yield and weather variable and n is the number of weeks. Indices with j = 0 are unweighted indices and j = 1 are weighted ones.

For this study, we have used n = 18, the value of i varies from 1 to 16, the value of i and i’ vary from 1 to 9. Following the above procedure, 90 weather indices were generated as presented in Table 1.

**Table 1:** Unweighted and weighted weather indices for the development of multivariate models.

Parameter	T_max_	T_min_	RH-I	RH-II	Rain	NOR/D	SSH	WV	E_vap_
T_max_	Z_1k_								
T_min_	Z_12k_	Z_2k_							
RH-I	Z_13k_	Z_23k_	Z_3k_						
RH-II	Z_14k_	Z_24k_	Z_34k_	Z_4k_					
Rain	Z_15k_	Z_25k_	Z_35k_	Z_45k_	Z_5k_				
NOR/D	Z_16k_	Z_26k_	Z_36k_	Z_46k_	Z_56k_	Z_6k_			
SSH	Z_17k_	Z_27k_	Z_37k_	Z_47k_	Z_57k_	Z_67k_	Z_7k_		
WV	Z_18k_	Z_28k_	Z_38k_	Z_48k_	Z_58k_	Z_68k_	Z_78k_	Z_8k_	
E_vap_	Z_19k_	Z_29k_	Z_39k_	Z_49k_	Z_59k_	Z_69k_	Z_79k_	Z_89k_	Z_9k_

k = 0,1 for unweighted/weighted indices respectively, *Tmax* maximum temperature, *Tmin* minimum temperature, *RH-I* relative humidity morning, *RH-II* relative humidity afternoon, *Rain* rainfall, *SSH* sunshine hours, *WV* wind velocity, *E*_*vap*_ Pan evaporation.

### Calculation of area under disease progress curve (AUDPC)

Area under Disease Progress Curve (AUDPC) is a quantitative measure of disease intensity with time. It was computed in Microsoft Excel using the following procedure^50^:

3 $$\:AUDPC={\sum\:}_{i=1}^{s}\frac{({Y}_{i}-{Y}_{i-1})}{2}*d$$

where *s* = No. of successive evaluations of disease; *i* = period; *Y*_*i*_ = disease severity at ith period; *Y*_*i−1*_ = disease severity at (*i*-1)^th^ period; *d* = time interval.

The Area Under Disease Progress Curve (AUDPC) was calculated using weekly disease severity data recorded throughout the soybean growing season. Disease severity was measured as a percentage (%). Before applying the classification models, the dataset underwent a thorough preprocessing phase. This included checking for outliers using the interquartile range (IQR) method, but no significant outliers were found, so no removal was necessary.

### Steps involved in model development

**Fig. 2:**
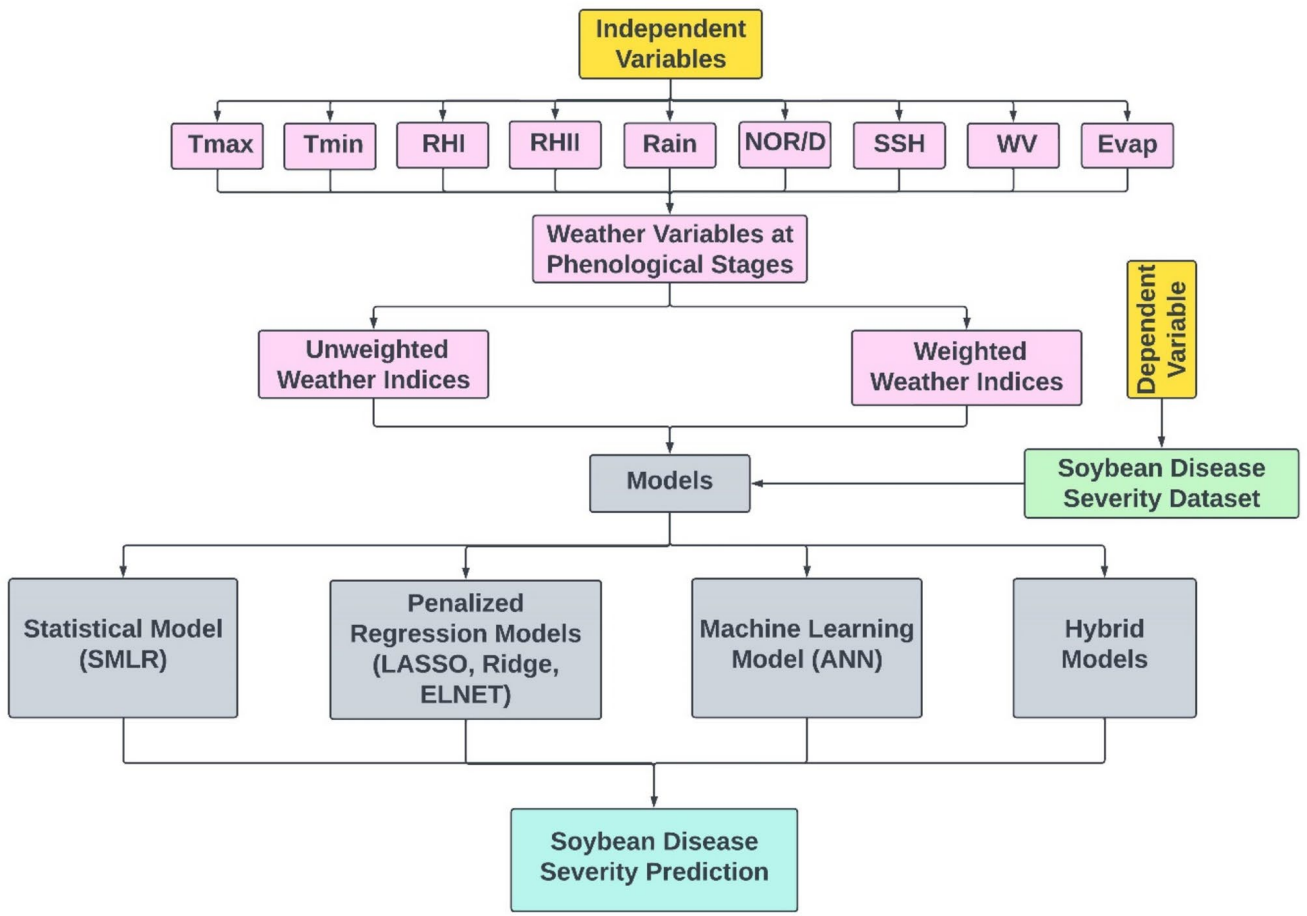
Steps involved in the model’s training and testing.

The steps involved in the model development are illustrated in Fig. 2. At the first step, average weather variables were used. These average values were then used to compute the weather indices. In the final step, these weather indices along with the disease data were used to form the forecast models by using different multivariate techniques.

### Multivariate techniques

We used six multivariate analysis methods, i.e., SMLR, LASSO, RR, ELNET, ANN and SMLR-ANN directly and with the combination of principal component analysis. The selection of modeling tools for predicting Soybean Yellow Mosaic Virus (SYMV) incidence was based on their ability to capture complex, nonlinear relationships between weather variables and disease severity. Traditional statistical models like Stepwise Multiple Linear Regression (SMLR) have been widely used for their interpretability and simplicity in identifying key predictors. However, they often struggle with multicollinearity and fail to account for intricate interactions among weather parameters. To address these limitations, regularization techniques such as LASSO, Ridge Regression (RR), and Elastic Net (ELNET) were incorporated to improve feature selection and model stability. All models in this study were implemented using R software (version R-4.4.3) and RStudio (version 2024.12.1–563). The software is available for download at: https://cran.rstudio.com/bin/windows/base/R-4.4.3-win.exe; https://posit.co/download/rstudio-desktop/.

Additionally, artificial neural networks (ANNs) were utilized due to their capacity to model nonlinear dependencies and detect hidden patterns in high-dimensional data. Hybrid models, particularly PCA-SMLR-ANN and PCA-ANN, were developed to leverage the strengths of both statistical and machine learning approaches, integrating principal component analysis (PCA) to reduce dimensionality and enhance predictive accuracy. Table 2 contains the details of tuned hyperparameters used by different models in this study.

**Table 2:** Tuned hyperparameters for different models.

Methods	Hyper parameter
PCA-SMLR	No. of PC’s: 11
ANN	Hidden layer sizes = 10, Max Iteration = 100, No. of PC’s: 11, decay = 0.451
PCA-ANN	Hidden layer sizes = 8, Max Iteration = 100, No. of PC’s: 11, decay = 0.301
LASSO	Alpha = 1 (L1 regularization)
PCA-LASSO	Alpha = 1 (L1 regularization)
RR	Alpha = 0 (L2 regularization)
PCA-RR	Alpha = 0 (L2 regularization)
ELNET	Alpha = 0,1 (Length = 10)
PCA-ELNET	Alpha = 0,1 (Length = 10)
SMLR-ANN	Hidden layer sizes = 5, Max Iteration = 100, No. of PC’s: 11, decay = 0.251
PCA-SMLR-ANN	Hidden layer sizes = 7, Max Iteration = 100, No. of PC’s: 11, decay = 0.401

#### Principal component analysis (PCA)

Principal component analysis (PCA) was applied to reduce the dimensionality of the dataset and address potential multicollinearity among weather indices. The analysis transformed the original set of weather variables into a smaller set of uncorrelated principal components (PCs) while retaining most of the dataset’s variability. In this study, only the principal components with eigenvalues greater than one were retained for further modeling, per the Kaiser criterion^51^. This approach ensured that the most significant patterns in the data were preserved while reducing redundant information^52^. The selected PCs were subsequently used as input variables in predictive models to assess their impact on forecasting SYMV severity.

#### Stepwise multiple linear regression (SMLR)

The SMLR technique, applied to the dataset comprising disease severity and weather variables, represents a straightforward method for building the disease severity forecast model. It entails a systematic approach to model construction, involving introducing or removing predictor variables. This method enables the identification of the most impactful predictors from a vast array of options^53,54^. Stepwise regression requires two critical thresholds: one for adding variables and another for removing variables. To prevent infinite loops, the probability threshold for adding variables must be lower than the threshold for removing variables^55^. This study used p-values of 0.50 and 0.10 as the thresholds for adding and removing variables, respectively. The ‘glmnet’ package was utilized to build a model employing Stepwise Multi Linear Regression (SMLR).

#### Penalized regression models (LASSO, RR and ELNET)

In scenarios where the dataset contains more variables than samples, traditional linear models often yield suboptimal results. To address this challenge, a more practical approach is employed through penalized regression, where the excess variables are constrained by introducing a penalty term to the equation. This reduction in the model’s dimensionality is commonly referred to as shrinkage or regularization. Regularization facilitates the adjustment of coefficients for less influential variables, often driving them closer to or even down to zero. The penalized regression techniques explored in this study encompass LASSO, RR and Elastic Net.

#### Least absolute shrinkage and selection operator (LASSO)

The LASSO method serves two primary functions: regularization and variable selection, rendering it a robust tool. Through a shrinking process, LASSO penalizes regression variable coefficients, causing some coefficients to diminish to zero. As a result, input variables with non-zero coefficients are chosen to be incorporated into the model. The main aim of LASSO is to minimize forecasting errors^56^. LASSO regression employs the L1 regularization technique. In this study, the implementation of LASSO was conducted using the ‘glmnet’ package within the R software environment^29,32,57^. The LASSO algorithm minimizes an objective function, which is expressed as follows:

4 $$\:{\text{L}}_{\text{l}\text{a}\text{s}\text{s}\text{o}}\:\left(\widehat{\beta\:}\right)=\sum\:_{\text{i}=1}^{\text{n}}{\left({\text{y}}_{\text{i}}-{\text{x}{\prime\:}}_{\text{i}}\widehat{{\upbeta\:}}\right)}^{2}+{\uplambda\:}\sum\:_{\text{j}=1}^{\text{m}}\left|\widehat{{{\upbeta\:}}_{\text{j}}}\right|$$

where ‘β’ represents the regression coefficient linked with the input parameters of the LASSO model. ‘x’ denotes the input, ‘y’ represents the output, and ‘n’ signifies the number of samples within the training dataset. The hyper-parameter ‘λ’ stands as the penalty parameter.

#### Ridge regression (RR)

Ridge regression is a method utilized to address overfitting in data by introducing a small amount of bias to the regression estimates. The primary objective of employing Ridge regression is to obtain more dependable estimates^29,32^. Ridge regression demonstrates consistent performance on both the training and testing datasets. It employs the L2 regularization technique. The loss function in ridge regression is defined as follows:

5 $$\:{\text{L}}_{\text{r}\text{i}\text{d}\text{g}\text{e}}\:\left(\widehat{\beta\:}\right)=\sum\:_{\text{i}=1}^{\text{n}}{\left({\text{y}}_{\text{i}}-{\text{x}{\prime\:}}_{\text{i}}\widehat{{\upbeta\:}}\right)}^{2}+{\uplambda\:}\sum\:_{\text{j}=1}^{\text{m}}{{\upbeta\:}}_{\text{j}}^{2\:\:}=\text{y}-\text{X}{\widehat{{\upbeta\:}}}^{2\:\:}+{\uplambda\:}{\widehat{{\upbeta\:}}}^{2\:\:}$$

where ‘x’ stands for the input vector, ‘y’ for the output vector, ‘n’ for the number of samples in the training dataset, ‘β’ for the regression coefficient, and ‘λ’ for the penalty parameter.

#### Elastic net (ELNET)

ELNET regression models integrate the strengths of both LASSO (Least absolute shrinkage and selection operator) and Ridge regression techniques, leveraging insights gained from the limitations of these models to enhance the overall performance of the final model^58,59^. In this approach, the optimization of two parameters, lambda and alpha, is performed. In ridge regression, alpha is assigned a value of 0 while in LASSO regression, it is set to 1. However, in ELNET, alpha can assume any value between 0 and 1. For the present study, the functioning of ELNET was carried out utilizing the ‘glmnet’ package within the R software environment^57^. The elastic net estimator minimizes the following expression:

6 $$\:{\text{L}}_{\text{e}\text{l}\text{n}\text{e}\text{t}}\:\left(\widehat{\beta\:}\right)=\frac{\sum\:_{\text{i}=1}^{\text{n}}{\left({\text{y}}_{\text{i}}-{\text{x}{\prime\:}}_{\text{i}}\widehat{{\upbeta\:}}\right)}^{2}}{2\text{n}}+\:{\uplambda\:}\left(\frac{1-{\upalpha\:}}{2}\sum\:_{\text{j}=1}^{\text{m}}{\widehat{{\upbeta\:}}}^{2}+{\upalpha\:}\sum\:_{\text{j}=1}^{\text{m}}\left|{\widehat{{\upbeta\:}}}_{\text{j}}\right|\right)$$

where ‘x’ represents the input, ‘y’ stands for the output, ‘n’ is the number of samples in the training dataset, ‘β’ denotes the regression coefficient, ‘λ’ is the penalty parameter, and ‘α’ serves as the mixing parameter that interpolates between ridge regression (α = 0) and LASSO (α = 1).

#### Artificial neural network (ANN)

ANN, or Artificial Neural Networks, are computational models inspired by the structure and function of the central nervous system^60–62^. Designed for machine learning tasks, these models are commonly depicted as interconnected systems of “neurons” capable of processing input data and computing values through the propagation of information across the network^63^. They comprise three layers: the input layer, the hidden layer and the output layer. During operation, data moves from the input layer through the hidden layer to the output layer^64^.

The input layer encompasses nodes representing both weighted and unweighted weather indices. The output layer encompasses the dependent variables, while the hidden layer hosts nodes associated with transfer (or activation) functions. Within each layer, neurons are connected to those in the subsequent layer through specific signals^62^. Each connection is assigned a weight. The final ANN output is computed by multiplying each input by its weight. This output then undergoes further processing through an activation function to yield the ultimate result of the neural network. The number of nodes in the input layer is depends on the quantity of independent predictors^31,65–67^. Every layer comprises interconnected neurons or nodes. The quantity of neurons in both the input and output layers is dictated by the dataset utilized. A key hurdle in implementing ANN is identifying the optimal number of hidden neurons or nodes. For this study, the number of hidden nodes was determined using the “train” function from the “caret” package in R software. The “nnet” method with 10-fold cross-validation was employed for this purpose^68^. The “tune grid” function was utilized to identify the optimal number of nodes in the hidden layer, aiming for the best model performance.

**Fig. 3:**
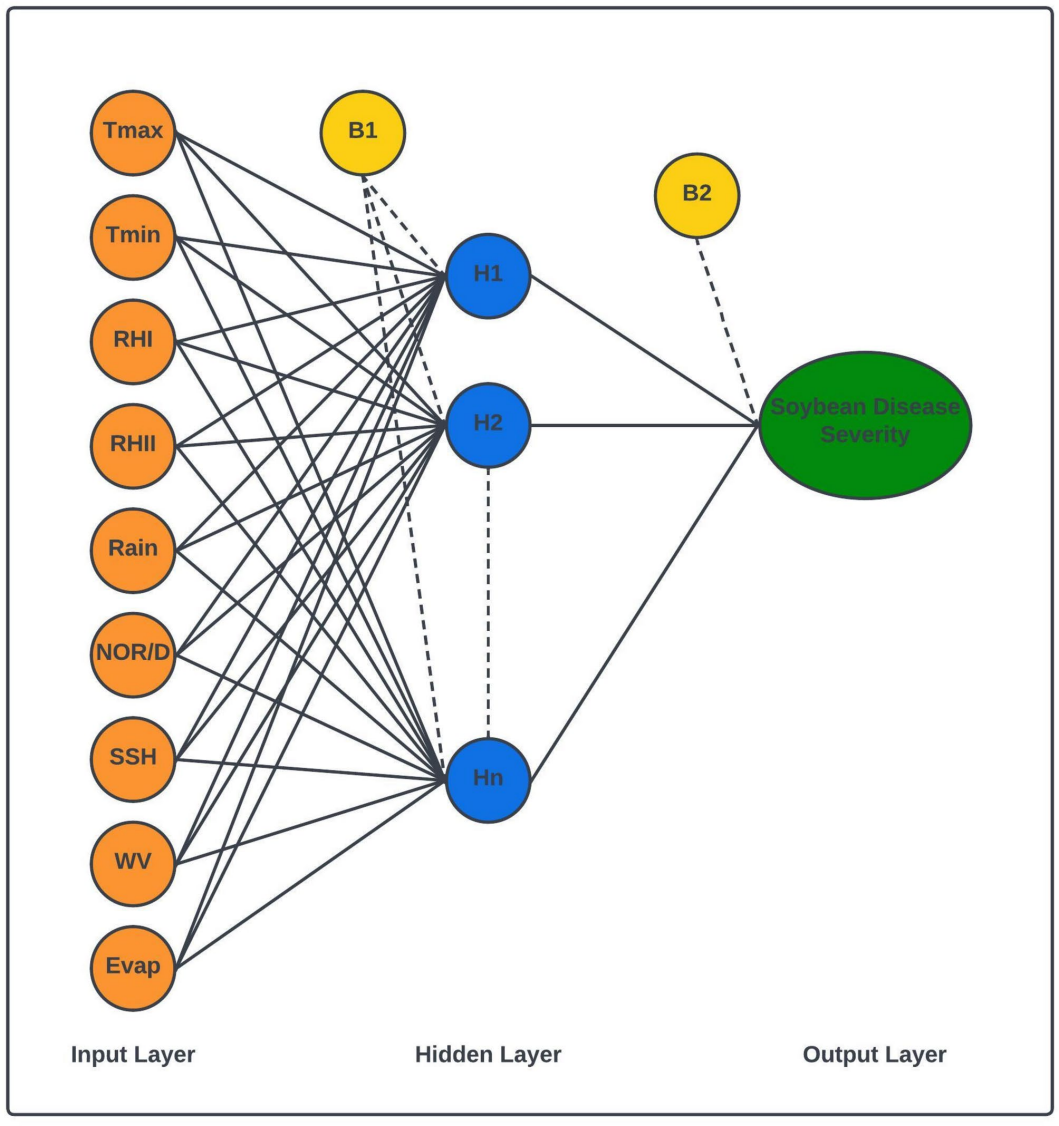
Flow diagram of the ANN model development.

Throughout the training phase of the Artificial Neural Network (ANN), a maximum iteration value of 100 was specified. Additionally, the package incorporated weight regularization via a hyperparameter termed “decay”. Weight decay acts as a regularization parameter intended to address potential overfitting issues in the neural network model trained on a specific dataset. Regularization in machine learning is analogous to shrinkage in Statistics, helping to prevent the model from becoming overly intricate and overly reactive to the training data. All weather indices were inputs, while disease severity served was the dependent variable (Fig. 3).

#### Hybrid models

The hybrid model that combines Stepwise Multiple Linear Regression (SMLR) with Artificial Neural Networks (ANN) offers a powerful approach for predictive modeling. In a standard neural network model, the initial weights and bias values assigned to the input layers are typically determined randomly^69^. In contrast, in a hybrid regression-neural network model, these initial weights and bias values supplied to the Artificial Neural Network (ANN) input layers are derived from a Stepwise Multiple Linear Regression (SMLR) equation. Specifically, the coefficients calculated for the independent variables within the SMLR equation are employed to initialize the input layer of the ANN model. This approach ensures that the neural network’s initial configuration is rooted in the relationships observed in the training data, providing a more data-informed starting point for the model.

In PCA-SMLR, PCA-LASSO, PCA-Ridge, PCA-ELNET, PCA-ANN and PCA-SMLR-ANN techniques, PCA scores were utilized as inputs for the analysis^70^. To mitigate the problem of multicollinearity among weather variables, Principal Component (PC) scores were employed as predictors for SMLR (Stepwise Multiple Linear Regression), LASSO (Least Absolute Shrinkage and Selection Operator), Ridge, and ELNET (Elastic Net), as well as for ANN (Artificial Neural Network) and SMLR-ANN (Stepwise Multiple Linear Regression-Artificial Neural Network) models, facilitating the construction of crop disease and yield models^71^. PCA (Principal Component Analysis) decomposes the original data matrix X into two matrices, P and T, represented as X = TP^t^. The matrix P is often termed the loading matrix, while the matrix T represents an orthogonal score matrix. The superscript T indicates the transpose of a matrix.

### Testing the performance of the models

The predictive accuracy of the models was evaluated using multiple statistical metrics, including the coefficient of determination (R²), root mean square error (RMSE), normalized root mean square error (nRMSE), mean absolute error (MAE), and percentage error (PE). Model performance was considered optimal when R² and efficiency factor (EF) approached 1, while RMSE and MAE values were minimized, and PE exhibited a narrow range. For comparative assessment, model performance was classified based on R² values as follows: R² > 0.90 indicated excellent performance, R² between 0.90 and 0.75 denoted good performance, R² between 0.75 and 0.50 represented fair performance, and R² < 0.50 signified poor performance^72,73^. Similarly, the nRMSE values were interpreted as follows: nRMSE < 10% corresponded to excellent performance, nRMSE between 10% and 20% indicated good performance, nRMSE between 20% and 30% reflected fair performance, and nRMSE > 30% was indicative of poor performance^38,49^. These were calculated using the formula given below:

7 $$\:{R}^{2}={\left[\frac{\frac{1}{n}\sum\:_{i=1}^{n}\left({y}_{i}-\stackrel{-}{y}\right)({\widehat{y}}_{i}-\widehat{\stackrel{-}{y}})}{{\sigma\:}_{y}{\sigma\:}_{\widehat{y}}}\right]}^{2}$$

8 $$\:RMSE=\sqrt{\frac{\sum\:_{i=1}^{n}{\left({y}_{i}-{\widehat{y}}_{i}\right)}^{2}}{n}}$$

9 $$\:nRMSE=\frac{100}{M}\sqrt{\frac{\sum\:_{i=1}^{n}{\left({y}_{i}-{\widehat{y}}_{i}\right)}^{2}}{n}}$$

10 $$\:MAE=\frac{\sum\:_{i=1}^{n}\left|{y}_{i}-{\widehat{y}}_{i}\right|}{n}$$

11 $$\:Percentage\:Error=\left(\frac{{y}_{i}-{\widehat{y}}_{i}}{{y}_{i}}\right)*100$$

where $\:{y}_{i}$ is the observed value, $\:{\widehat{y}}_{i}$ is the predicted value for i = 1, 2, 3,…, n. $\:\stackrel{-}{y}$ and $\:{\widehat{y}}_{i}$ denote the average of the observed and predicted values, respectively. $\:{\sigma\:}_{y}$ and $\:{\sigma\:}_{\widehat{y}}$ are the standard deviations of observed and predicted observations. *M* is the mean of the observed variables and n is the total number of observations.

## Results and discussion

### Characteristics of soybean yellow mosaic virus

**Fig. 4:**
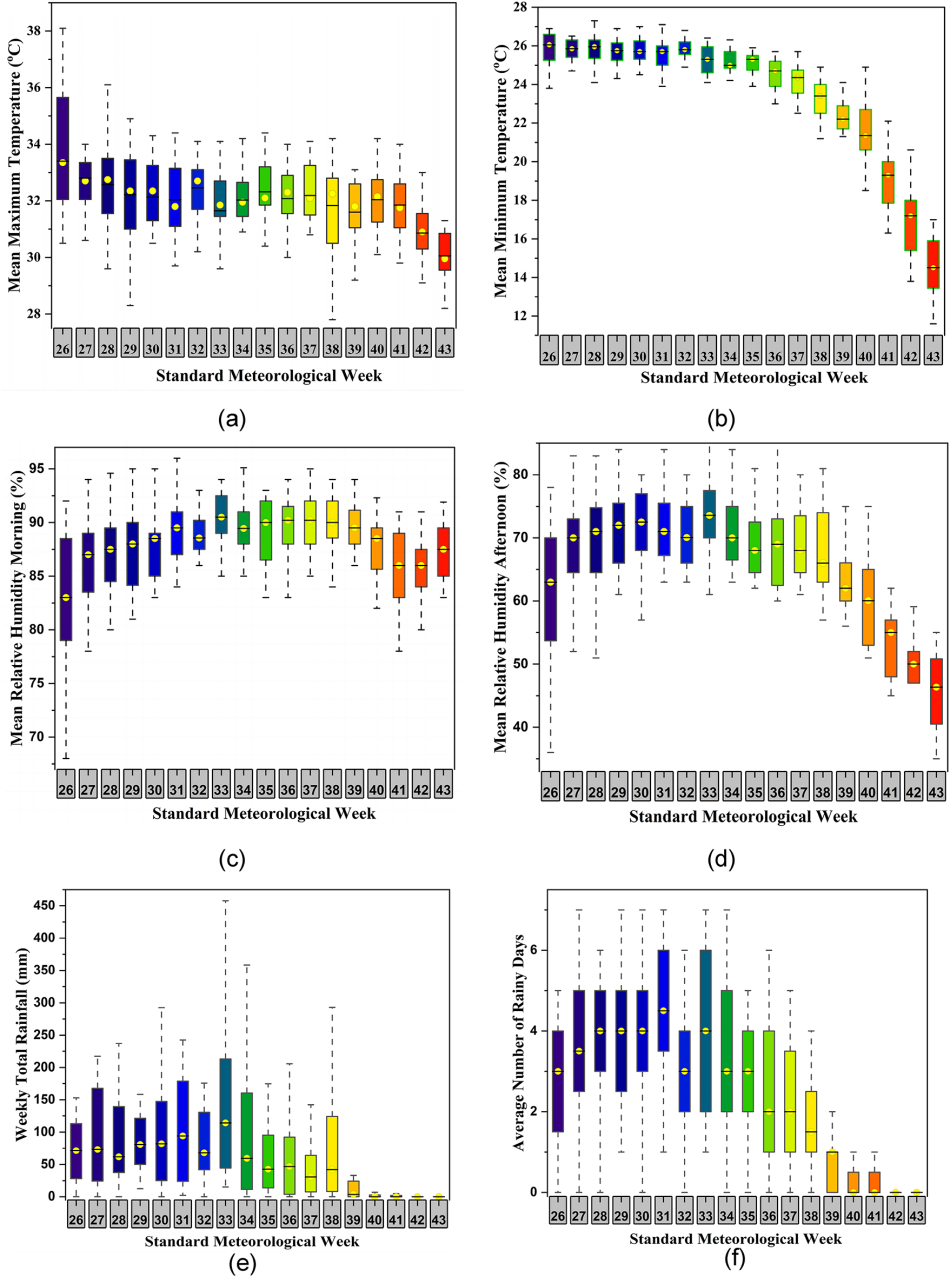

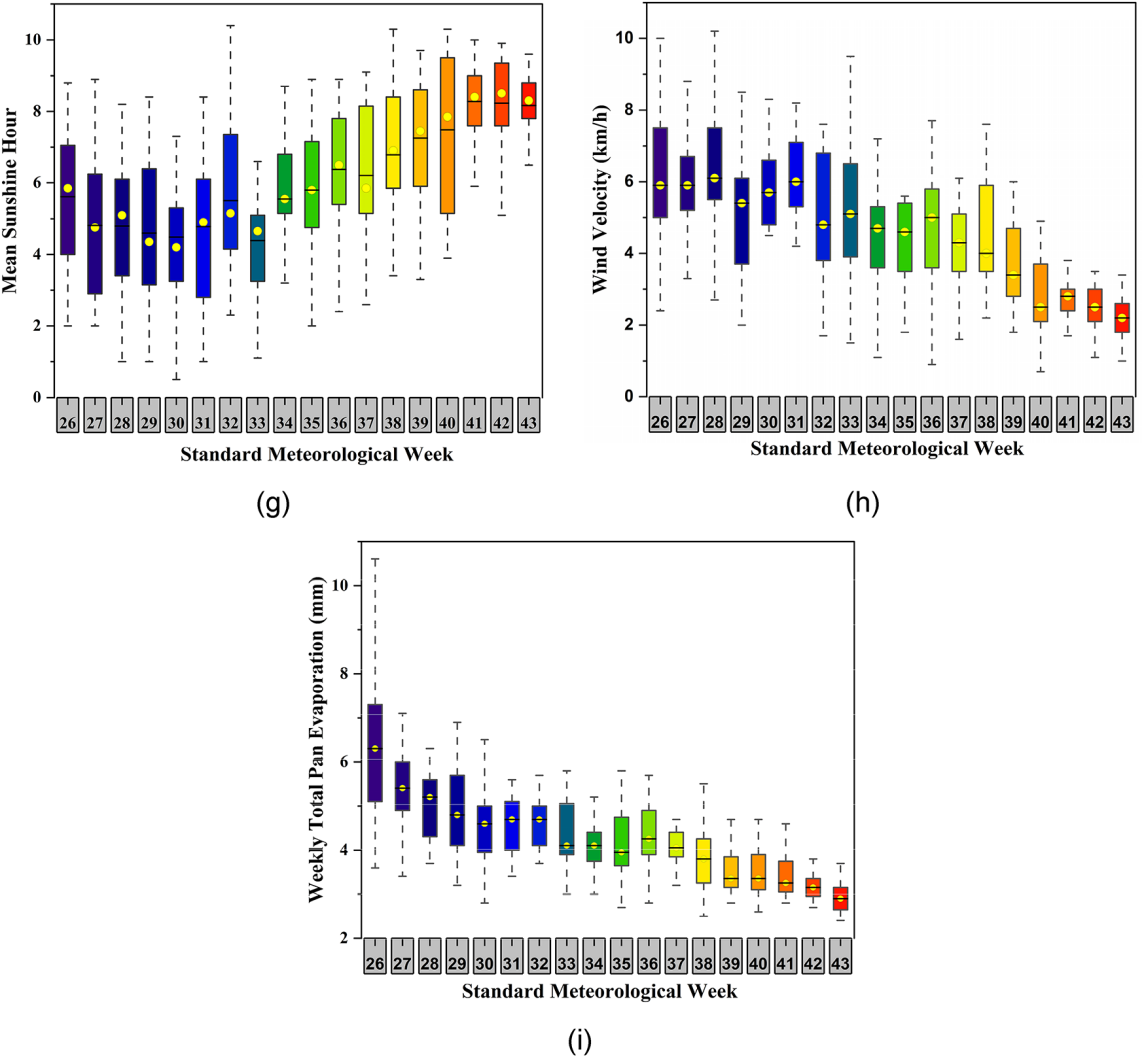
Boxplot of the nine input variables during the soybean growth (2001–2020) (**a**) maximum temperature, (**b**) minimum temperature, (**c**) relative humidity morning, (**d**) relative humidity afternoon, (**e**) total rainfall, (**f**) number of rainy days, (**g**) sunshine hour, (**h**) wind velocity and (**i**) pan evaporation. *Standard Meteorological Weeks (SMWs) 26 to 43 are included because this period corresponds to the soybean growing season in the study region.

A comprehensive review of past literature reveals intricate associations between weather variables and the dynamics of soybean mosaic virus (SMV) across various geographical regions. Aravind et al.^74^ underscored the pivotal role of minimum temperature, evening relative humidity, rainfall, and sunshine hours in the development of SMV, particularly in the Tarai region of Uttarakhand. Their findings highlight the nuanced interplay between temperature, humidity, and precipitation levels during specific stages of disease transmission, elucidating critical thresholds for optimal virus proliferation. Furthermore, the study conducted by Nagamani et al.^75^ provided valuable insights into the significant influence of temperature on the intensity of soybean mosaic virus (SMV), revealing distinct temperature ranges that affect disease occurrence among leguminous crops. Moreover, it was found that maximum temperature, sunshine hours positively affected the disease incidence and rainfall, evening relative humidity were negatively related. This finding was contradicted by Suman et al.^76^, who highlighted a significant and positive correlation between the relative humidity, rainfall and disease incidence, as well as a negative influence of maximum temperature on SMV dissemination. Noteworthy is study done by Khan et al.^77^, which underscored the synergistic impact of temperature, relative humidity, and rainfall on SMV infections, emphasizing the dynamic interaction of multiple weather variables in shaping disease dynamics. Taken together, these findings underscore the multifaceted influence of weather conditions on SMV epidemiology, providing a cornerstone for future research endeavors aimed at unraveling the intricate mechanisms governing virus transmission and guiding targeted disease management strategies in agricultural contexts. Figure 4 demonstrates the boxplots of all nine different input variables during the soybean growth (2001–2020) over the study region (a) maximum temperature, (b) minimum temperature, (c) Relative humidity morning, (d) relative humidity afternoon, (e) Total rainfall, (f) Number of rainy days, (g) Sunshine hour, (h) Wind velocity and (i) Pan evaporation.

### Performance of SMLR and PCA-SMLR

The values of prediction accuracy statistics of all models are given in Table 3. Initially the performance of Stepwise Multiple Linear Regression Model based on weather indices was evaluated. The coefficient of determination (R^2^ was 0.81, signifying that around 81% of the variation in soybean disease severity was caused by the significant predictors (Z_691_ and Z_791_). The root mean square error (RMSE) during calibration was 27.65, while during validation, it was 119.88. The value of nRMSE during calibration was 14.91% and that during validation was 47.72%. MAE at the calibration stage and validation stage were found to be 23.68 and 100.37, respectively. A decrease in R^2^ value and an increase in errors (RMSE, nRMSE and MAE) during the validation were observed. The SMLR model did not perform consistently during the calibration and validation. The error percentage ranged from − 87.43 to 63.58% (Table S1).

**Table 3:** Quantitative measures obtained of different models during calibration and validation.

Model	*R* ^2^	MAE	RMSE	nRMSE	*R* ^2^	MAE	RMSE	nRMSE
	Calibration				Validation			
SMLR	0.81	23.68	27.65	14.91	0.07	100.37	119.88	47.72
PCA-SMLR	0.85	21.38	24.89	13.42	0.45	101.14	110.82	44.72
ANN	1.00	4.70	5.71	3.08	0.99	11.58	17.12	6.82
PCA-ANN	0.99	2.28	2.77	1.49	1.00	7.74	9.21	3.67
LASSO	0.97	11.62	12.99	7.01	0.84	105.14	118.53	47.19
PCA-LASSO	0.84	27.07	30.12	16.25	0.78	98.19	108.98	43.38
RR	0.90	22.88	26.68	14.39	0.84	92.33	108.47	43.18
PCA-RR	0.93	21.02	26.88	14.50	0.01	102.28	115.36	45.92
ELNET	0.90	22.88	26.68	14.39	0.84	92.33	108.47	43.18
PCA-ELNET	0.95	12.71	15.22	8.21	0.15	108.31	118.23	47.07
SMLR-ANN	0.97	5.52	11.74	6.33	0.96	4.69	5.11	2.22
PCA-SMLR-ANN	1.00	2.80	5.27	2.84	0.99	1.34	1.59	0.76

**Fig. 5:**
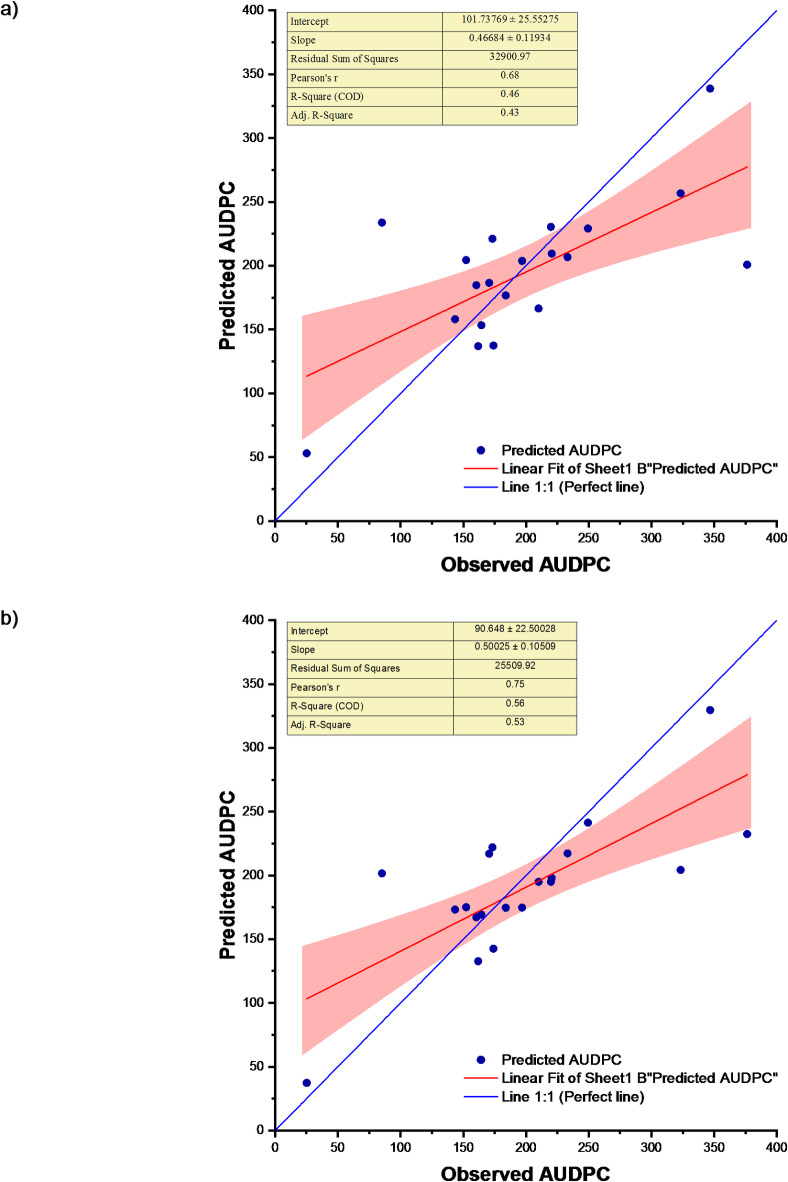
Observed and predicted AUDPC for (**a**) SMLR and (**b**) PCA-SMLR models.

After this, the Principal Component Analysis-Stepwise Multiple Linear Regression (PCA-SMLR) Model utilizing weather indices was developed for the prediction of soybean disease severity, in which case, the coefficient of determination (R^2^ was 0.85. This suggests that the significant predictors accounted for approximately 85% of the variation in soybean disease severity (PC1, PC3, and PC4). The root mean square error (RMSE) during calibration was determined to be 24.89, whereas during validation, it was observed to be 110.82. nRMSE value during calibration was 13.42% and that of validation was 44.72%. MAE at calibration and validation stages were found 21.38 and 101.14, respectively. Decrease in R^2^ value and increase in errors (RMSE, nRMSE and MBE) during validation were observed. PCA-SMLR model performed consistently during calibration and validation. Table 3 reveals that the PCA-SMLR model better fits the data compared to the SMLR model for predicting soybean disease severity. The error percentage ranged from − 62.04 to 57.76% (Table S1). The scatter plot of SMLR and PCA-SMLR models observed and predicted disease severity for the study location is shown in Fig. 5.

### Performance of ANN and PCA-ANN

Artificial Neural Network (ANN) Model was fitted to the data of soybean disease severity and weather indices. Our experimental findings observed that an Artificial Neural Network (ANN) and PCA-ANN processes input by computing the weighted sum of inputs while integrating an adjustable bias. For the artificial neural network model based on weather indices, the prediction accuracy, as indicated by the coefficient of determination (R^2^ value and root mean square error (RMSE) during calibration, was found to be 1.00 and 5.71, respectively. During validation, the R^2^ value was 0.99, and the RMSE value was 17.12. The normalized RMSE (nRMSE) values during calibration and validation were found to be 3.08% and 6.82%, respectively. The values of MAE at the calibration and validation stages were found to be 4.70 and 11.58 respectively. An increase in the value of R^2^ and errors (RMSE, nRMSE and MAE) during validation were observed in this methodology. The model exhibited excellent performance during both the calibration and validation stages. The error percentage ranged from 0.33 to 8.71% (Table S2).

**Fig. 6:**
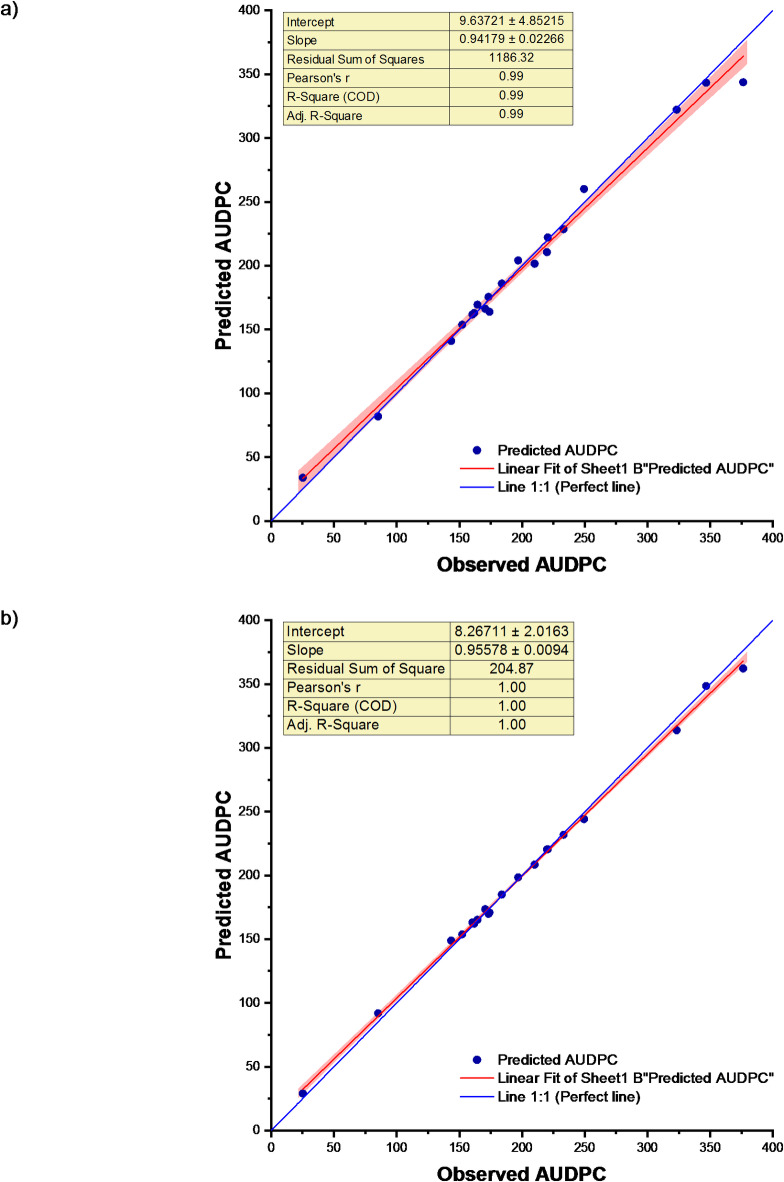
Observed and predicted AUDPC for (**a**) ANN and (**b**) PCA-ANN models.

Further, the Principal Component Analysis-Artificial Neural Network Model was fit to the data by using the principal components. The performance of the Principal Component Analysis-Artificial Neural Network Model based on weather indices (PCA-ANN) demonstrated an excellent approximation. The coefficient of determination (R^2^ was 0.99, and the root mean square error (RMSE) during calibration was 2.77. During validation, the R^2^ value was 1.00, and the RMSE value was 9.21. The normalized RMSE (nRMSE) values during calibration and validation were 1.49% and 3.67%, respectively. MAE at the calibration stage and validation stage was found to be 2.28 and 7.74, respectively. An increase in R^2^ value and errors (nRMSE and MAE) during validation were observed. The error percentage varied from − 7.91 to 3.79% (Table S2). The size and decay for ANN were determined to be (10, 0.451), while for PCA-ANN, they were found to be (8, 0.301), as illustrated in Figs. S1 and S2. The scatter plots of ANN and PCA-ANN models for observed and predicted disease severity are shown in Fig. 6. From Table 3, it can be observed that PCA-ANN (Hybrid) model is a better model than the ANN (Individual) model.

### Performance of LASSO and PCA-LASSO

In this section, we have fitted the Least Absolute Shrinkage and Selection Operator (LASSO) and Principal Component Analysis Least Absolute Shrinkage and Selection Operator (PCA-LASSO) Models to the data of soybean disease severity and weather indices. Initially, the performance of the Least Absolute Shrinkage and Selection Operator (LASSO) Model based on weather indices (LASSO) was assessed. The coefficient of determination (R^2^ value was 0.97. The root mean square error (RMSE) during calibration was 12.99, while during validation, it was 118.53. The normalized RMSE (nRMSE) value during calibration was 7.01%, and during validation, it was 47.19%. MAE at calibration and validation stages were found to be 11.62 and 105.14 respectively. A decrease in R^2^ value and an increase in errors (RMSE, nRMSE and MAE) during validation were observed. The LASSO model was performed consistently during calibration and validation. Error percentage ranged from − 134.30 to 40.07% (Table S3).

**Fig. 7:**
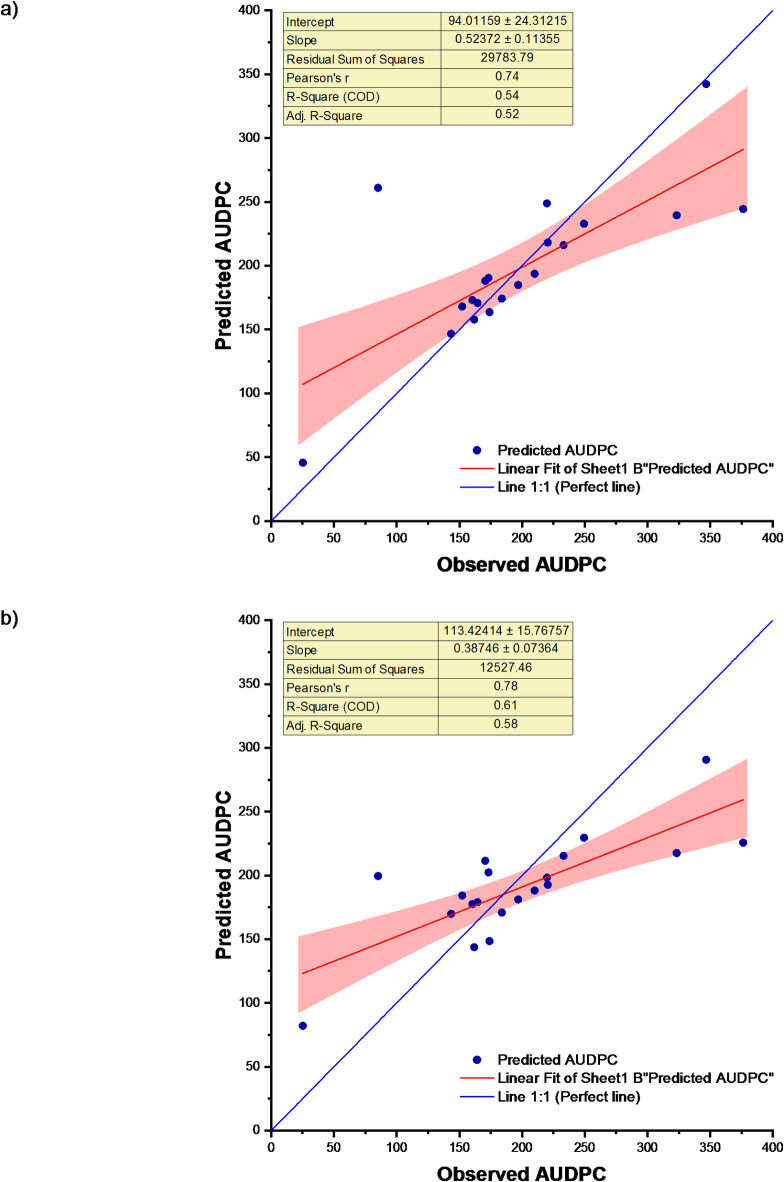
Observed and predicted AUDPC for (**a**) LASSO and (**b**) PCA-LASSO models.

In the Principal Component Analysis-Least Absolute Shrinkage and Selection Operator Model based on weather indices (PCA-LASSO), the coefficient of determination (R^2^ value was 0.84. The root mean square error (RMSE) during calibration was 30.12, while during validation, it was 108.98. The normalized RMSE (nRMSE) value during calibration was 16.25%, and during validation, it was 43.38%. MAE at the calibration stage and validation stage was found to be 27.07 and 98.19, respectively. A decrease in R^2^ value and an increase in errors (RMSE, nRMSE and MAE) during validation were observed. The PCA-LASSO model was performed consistently during calibration and validation. Error percentage ranged from − 144.87 to 63.98% (Table S3). The scatter plots of LASSO and PCA-LASSO models for observed and predicted disease severity for the study location are shown in Fig. 7. It can be observed from Table 3 that the LASSO model is a better fit than the PCA-LASSO model for this data.

### Performance of RR and PCA-RR

In this section, we have fitted Ridge Regression (RR) and Principal Component Analysis-Ridge Regression (PCA-RR) Models to the data of soybean disease severity and weather indices. For the Ridge Regression model, the coefficient of determination (R^2^ and root mean square error (RMSE) during calibration were 0.90 and 26.68, respectively. During validation, the R^2^ value was 0.84, and the RMSE value was 108.47. The normalized RMSE (nRMSE) values during calibration and validation were found to be 14.39% and 43.18%, respectively. MAE at the calibration stage and validation stage was found to be 22.28 and 92.33, respectively. A decrease in R^2^ value and an increase in errors (RMSE, nRMSE and MAE) during validation were observed. The performance of the model was good during calibration but poor during validation. Error percentage ranged from − 152.35 to 39.30% (Table S4).

**Fig. 8:**
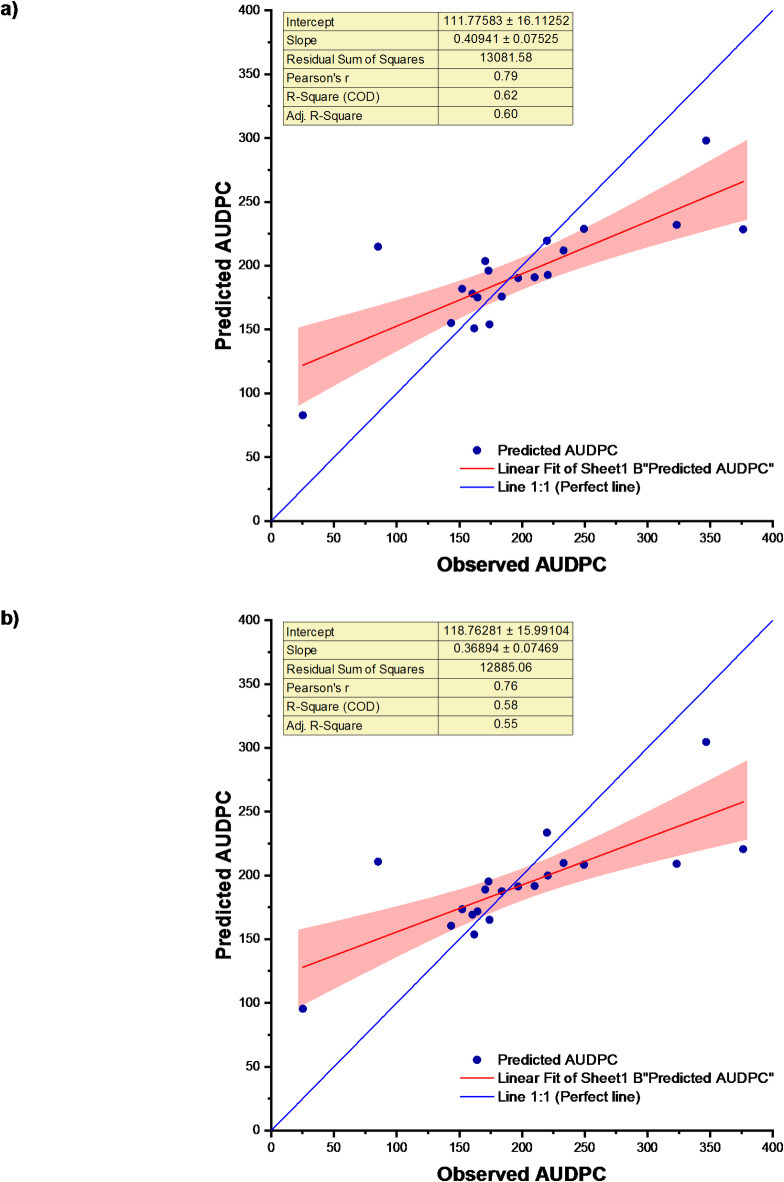
Observed and predicted AUDPC for (**a**) RR and (**b**) PCA-RR models.

For the Principal Component Analysis-Ridge Regression (PCA-RR) Model, the coefficient of determination (R^2^ and root mean square error (RMSE) during calibration were determined to be 0.93 and 26.88, respectively. During validation, the R^2^ value was 0.01, and the RMSE value was 115.36. The normalized RMSE (nRMSE) values during calibration and validation were found to be 14.50% and 45.92%, respectively. MAE at the calibration stage and validation stage were found to be 21.02 and 102.28, respectively. Error percentage ranged from − 147.44 to 41.35% (Table S4). The scatter plot of RR and PCA-RR models for observed and predicted disease severity for the study location is shown in Fig. 8. Table 3 exhibits that the PCA-RR model is a better fit than the RR model to this data at the calibration stage, but it is inferior to the RR model at the validation stage.

### Performance of ELNET and PCA-ELNET

In this section, ELNET and PCA-ELNET models have been fitted to the data of soybean disease severity and weather indices. The predicted accuracy, as indicated by the coefficient of determination (R^2^ value and root mean square error (RMSE) value during calibration, was determined to be 0.90 and 26.68, respectively, for the elastic net model based on weather indices. During validation, the R^2^ value was 0.84, and the RMSE value was 108.47. The normalized root mean square error (nRMSE) values during calibration and validation were found to be 14.39% and 43.18% respectively. MAE at calibration stage and validation stage was found to be 22.88 and 92.33 respectively. Decrease in R^2^ value and increase in errors (RMSE, nRMSE and MAE) during validation were observed. The performance of the model was good during calibration but poor during validation. Error percentage ranged from − 152.35 to 39.30% (Table S5).

**Fig. 9:**
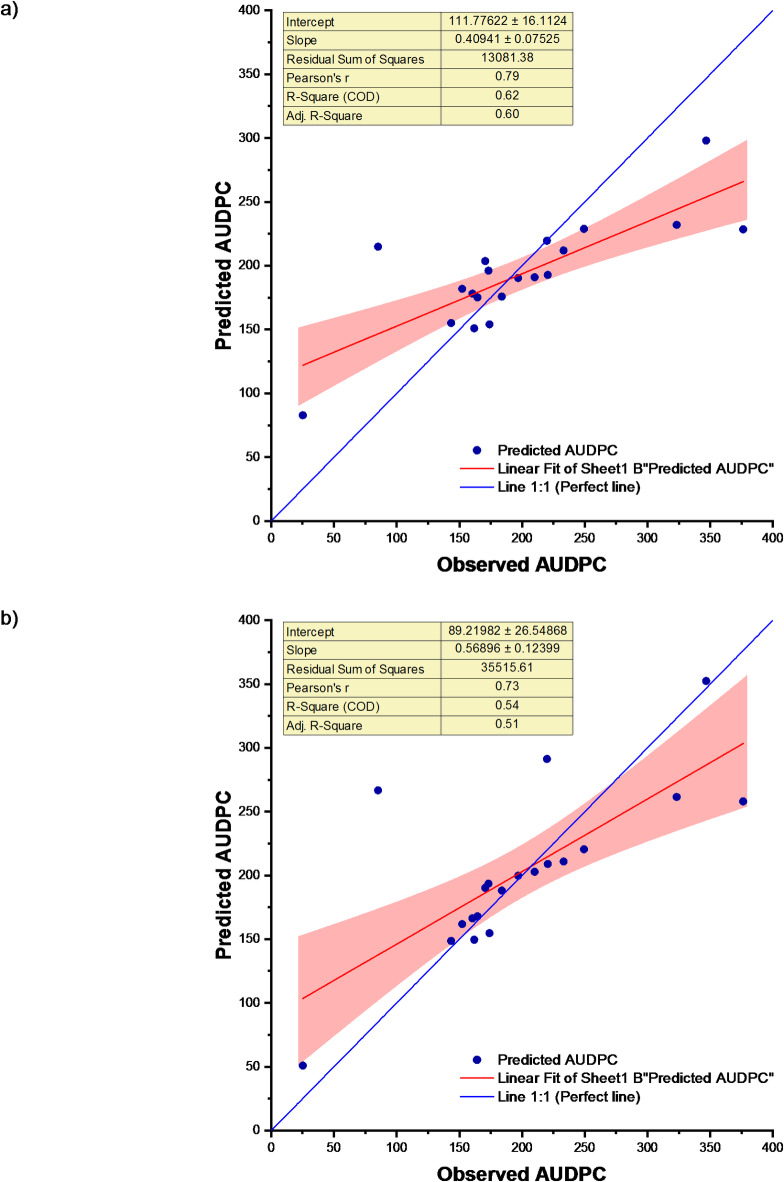
Observed and predicted AUDPC for (**a**) ELNET and (**b**) PCA-ELNET models.

In the Principal Component Analysis-Elastic Net (PCA-ELNET) model based on weather indices, the coefficient of determination (R^2^ and root mean square error (RMSE) during calibration were determined to be 0.95 and 15.22, respectively. During validation, the R^2^ value was 0.15, and the RMSE value was 118.23. The normalized RMSE (nRMSE) values during calibration and validation were found to be 8.21% and 47.07%, respectively. MAE values at the calibration and validation stages were found to be 12.71 and 108.31, respectively. A decrease in R^2^ value and an increase in errors (RMSE, nRMSE and MAE) during validation were observed. The models demonstrated excellent performance during the calibration stage but exhibited poor performance during the validation stage. The error percentage ranged from − 213.20 to 31.47% (Table S5). The scatter plot of ELNET and PCA-ELNET models for observed and predicted disease severity for the study location is shown in Fig. 9. Table 3 shows that the ELNET model may be considered a better fit than PCA-ELNET model. This may be because PCA condenses information of several weather indices into their linear combinations, which are called principal components.

### Performance of SMLR-ANN and PCA-SMLR-ANN

In this section, SMLR-ANN and PCA-SMLR-ANN models have been fitted to the data of soybean disease severity and weather indices. In our experimental findings, it was observed that a Stepwise Multiple Linear Regression-Artificial Neural Network (SMLR-ANN) and PCA-SMLR-ANN processes input by computing the weighted sum of inputs while integrating an adjustable bias. For SMLR-ANN model, the value of Coefficient of determination (R^2^ and RMSE during calibration was found to be 0.97 and 11.74, respectively. R^2^ value during validation was found to be 0.96 with RMSE value of 5.11. The nRMSE value during the calibration and validation stages was found to be 6.33% and 2.22%, respectively. MAE values at the calibration and validation stages were found to be 5.52 and 4.69, respectively. A decrease in R^2^ value and errors (RMSE, nRMSE and MAE) during validation were observed. The model’s performance was excellent during both calibration and validation stage. Error percentage ranged from − 3.12 to 1.00% (Table S6).

**Fig. 10:**
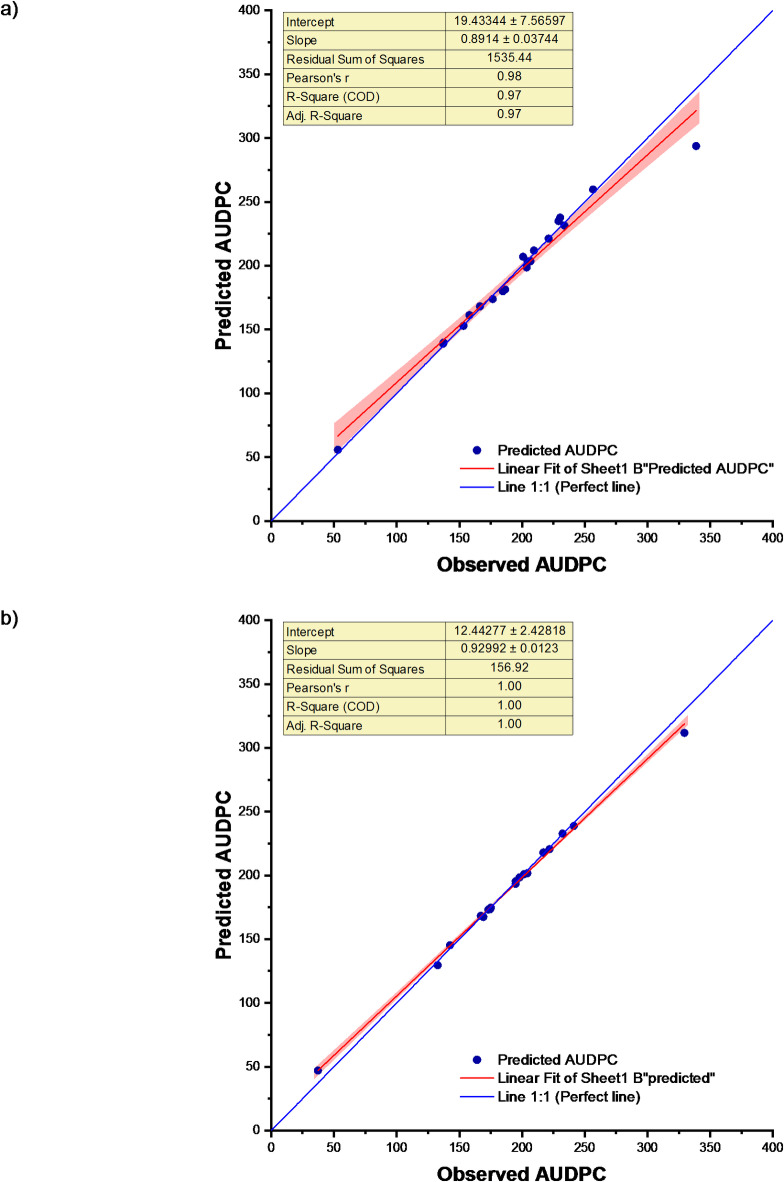
Observed and predicted AUDPC for (**a**) SMLR-ANN and (**b**) PCA-SMLR-ANN.

The prediction accuracy indicated by Principal Component Analysis-Stepwise Multiple Linear Regression-Artificial Neural Network (PCA-SMLR-ANN) model has been shown in Table 3. The Coefficient of determination (R^2^ and RMSE values during calibration were found to be 1.00 and 5.27, respectively. R^2^ value during validation was found to be 0.99 with RMSE value of 1.59. The nRMSE value during calibration and validation was found to be 2.84% and 0.76% respectively. MAE values at calibration and validation stages were found to be 2.80 and 1.34 respectively. Decrease in R^2^ value and errors (RMSE, nRMSE and MAE) during validation were observed. Error percentage ranged from − 2.62 to 2.13% (Table S6). Size and decay for SMLR-ANN and PCA-SMLR-ANN were found to be (5, 0.251) and (7, 0.401) respectively (Figs. S3, S4). The scatter plot of SMLR-ANN and PCA-SMLR-ANN models for observed and predicted disease severity for the study location is shown in Fig. 10. Table 3 exhibits that PCA-SMLR-ANN (a hybrid model with PCA) is a better model than the SMLR-ANN model (a hybrid model without PCA).

## Discussion

In recent years, integrating weather parameters with machine learning models for plant disease detection across various crops has gained significant importance. Advanced ML, AI and DL techniques are instrumental in managing agricultural pests, providing a robust strategy for protecting trees from various diseases, especially those affecting the leaves. Systematic identification of diseases in soybean disease severity, beginning at the initial infection stage, is crucial for effectively addressing this issue within the agricultural sector. This study aims to introduce a novel and compare the compared the impact of weather indices on disease severity. A comprehensive dataset encompassing both diseased and healthy soyabean crop states is essential for conducting this research effectively.

In plant disease identification, numerous studies have employed a deep hybrid approach, leveraging datasets from sources like Plant Village, both public and private. These investigations have achieved notable accuracies, typically ranging from 81.0 to 100.0% (R^2^_cal_, R^2^_val_, nRMSE_cal_, and nRMSE_val_)^78,79^. All developed models’ performance was compared using R² and nRMSE values, as presented in Table 4. According to these metrics, the ANN, PCA-ANN, LASSO, PCA-RR, PCA-ELNET, SMLR-ANN, and PCA-SMLR-ANN models demonstrated excellent performance during the calibration phase. Conversely, the SMLR, PCA-SMLR, PCA-LASSO, RR, and ELNET models exhibited satisfactory performance. During the validation phase, the ANN, PCA-ANN, SMLR-ANN, and PCA-SMLR-ANN models maintained their excellent performance, while the LASSO, PCA-LASSO, RR, PCA-RR, ELNET, and PCA-ELNET models showed satisfactory results. However, the SMLR and PCA-SMLR models did not perform satisfactorily during this stage. Based on the R² and nRMSE values, the PCA-SMLR-ANN model emerged as the most effective for predicting soybean disease severity in the study region, outperforming other models that also performed excellently, such as ANN, PCA-ANN, and SMLR-ANN.

The ranking of the models based on their overall performances and scores can be provided as follows: PCA-SMLR-ANN ≈ PCA-ANN ≈ SMLR-ANN ≈ ANN > PCA-ELNET > PCA-Ridge > ELNET ≈ RR > PCA-LASSO > LASSO > PCA-SMLR ≈ SMLR. These findings also confirm the conclusions of the study carried out by Singh and Nain^80^ and Alves et al.^81^ in which case the performance of ANN was better as compared to other models. Besides, this study also shows the superior performance of hybrid PCA-SMLR-ANN model over the individual models.

**Table 4:** Comparison of the models based on R^2^ and nRMSE values.

Model	*R* ^2^_cal_	*R* ^2^_val_	nRMSE_cal_	nRMSE_val_	Overall performance
SMLR	Good (0.81)	Poor (0.07)	Good (14.91)	Poor (47.72)	Poor
PCA-SMLR	Good (0.85)	Poor (0.45)	Good (13.42)	Poor (44.72)	Poor
ANN	Excellent (1.00)	Excellent (0.99)	Excellent (3.08)	Excellent (6.82)	Excellent
PCA-ANN	Excellent (0.99)	Excellent (1.00)	Excellent (1.49)	Excellent (3.67)	Excellent
LASSO	Excellent (0.97)	Good (0.84)	Excellent (7.01)	Poor (47.19)	Fair
PCA-LASSO	Good (0.84)	Good (0.78)	Good (16.25)	Poor (43.38)	Good
RR	Good (0.90)	Good (0.84)	Good (14.39)	Poor (43.18)	Good
PCA-RR	Excellent (0.93)	Poor (0.01)	Good (14.50)	Poor (45.92)	Good
ELNET	Good (0.90)	Good (0.84)	Good (14.39)	Poor (43.18)	Good
PCA-ELNET	Excellent (0.95)	Poor (0.15)	Excellent (8.21)	Poor (47.07)	Good
SMLR-ANN	Excellent (0.97)	Excellent (0.96)	Excellent (6.33)	Excellent (2.22)	Excellent
PCA-SMLR-ANN	Excellent (1.00)	Excellent (0.99)	Excellent (2.84)	Excellent (0.76)	Excellent

R^2^_cal_ and R^2^_val_ = Coefficient of determination during the calibration and validation, nRMSE_cal_ and nRMSE_val_= Normalized root mean square error during the calibration and validation, respectively.

Malik et al.^79^ propose a hybrid approach for classifying sunflower leaf diseases using deep learning techniques, focusing on four diseases: Alternaria leaf blight, downy mildew, Phoma blight, and Verticillium wilt. The proposed hybrid model outperformed existing deep learning models in terms of accuracy, achieving an accuracy of 89.2% on the same dataset used for comparison. Ardalkar et al.^33^ proposed a novel approach for image-based olive leaf diseases classification involves utilizing deep learning techniques, particularly a hybrid model that combines handcrafted features with learned features. El Akhal et al.^82^ presented a novel method for classifying olive leaf diseases, which integrates deep learning architectures, particularly convolutional neural networks (CNNs), with machine learning classifiers for image-based disease classification. The study’s findings indicated that the optimal deep hybrid model was achieved by integrating the EfficientNetB0 model with a logistic regression classifier, resulting in a remarkable accuracy score of 96.14%. Singh et al.^83^ employed thermal and visible imaging with machine learning (ML) and model combination (MC) techniques to predict chickpea wilt severity. The results of this study demonstrate that combining model combination (MC) techniques with machine learning (ML) models significantly enhances the accuracy of predicting plant disease severity under field conditions. Seyedmohammadi et al.^84^ used C&RT, k-NN and SVM as benchmark models in analyzing relationships between Pistachio yield and soil properties. Based on the findings, the k-nearest neighbors, classification and regression tree, and support vector machines algorithms individually explained 83%, 84%, and 88% of the variation in pistachio yield, respectively. However, the hybrid model significantly improved this predictive capability to 94%. This enhancement can be attributed to the hybrid model’s enhanced capacity to capture complex non-linear relationships more effectively.

The outcomes of this study will contribute to advancing agricultural disease prediction methodologies, helping farmers and policymakers implement more precise and timely disease management interventions. However, most existing studies have been constrained to detecting plant diseases using machine learning models under controlled environmental conditions. Apart from this, the MC techniques were also not tested under such conditions. Early detection of diseases during the growth stages of agricultural products is crucial, akin to its importance in human health. Prompt identification of illnesses can enable farmers to implement timely precautions, thereby reducing economic losses and preserving nutritional yield. Our study underscores the gap in the literature, where research on soybean disease severity prediction often focused on identification post-manifestation. To address this limitation, we propose future research aimed at predictive modeling to anticipate diseases before their onset, potentially enhancing proactive disease management strategies.

## Conclusions

In conclusion, early detection of plant diseases is critical for safeguarding crops, given their role as the primary cause of global agricultural losses and substantial economic damage. In this study, we developed several models, including SMLR, ANN, LASSO, RR, ELNET, and SMLR-ANN, to predict soybean disease severity using weather indices derived from weather variables. PCA-SMLR, PCA-ANN, PCA-LASSO, PCA-RR, PCA-ELNET, and PCA-SMLR-ANN models were developed by applying principal component analysis (PCA) to weather index data. These models were comprehensively compared to identify the optimal model for soybean disease severity prediction. Our findings indicate that the PCA-SMLR-ANN hybrid model exhibits superior performance in predicting disease severity across diverse conditions, demonstrating higher predictive accuracy compared to other models evaluated. This hybrid approach leverages PCA for feature reduction and combines strengths from SMLR and ANN algorithms, enhancing its efficacy in agricultural forecasting.

## Limitations and future scope

While the developed models demonstrated high predictive accuracy, certain limitations must be acknowledged. One key limitation is the potential variability in soybean varieties, which may exhibit different responses to weather conditions and disease susceptibility. Since the dataset primarily represents soybean crops grown in Pantnagar, Uttarakhand, the findings may not be fully generalizable to other regions with different climatic conditions, soil types, and management practices. Additionally, the models rely on historical weather data, which may not capture sudden environmental fluctuations, extreme weather events, or microclimatic variations that can influence disease dynamics. The effectiveness of hybrid models, though superior in this study, could also be affected by data quality, feature selection techniques, and computational complexity. Future research can enhance these models by incorporating real-time weather monitoring, remote sensing data, and genetic information related to soybean disease resistance.

Future studies could further explore the robustness of the PCA-SMLR-ANN model under varying environmental conditions and extend its application to different crops or regions. Additionally, investigations into integrating real-time data streams and advanced machine learning techniques could enhance the model’s predictive capabilities and practical utility in agricultural management and decision-making processes. These advancements promise to optimize disease management strategies and improve crop yield predictions in agricultural settings.

## Electronic supplementary material

Below is the link to the electronic supplementary material.

Supplementary Material 1

## Data Availability

The data can be shared on reasonable request from the corresponding author Dinesh Kumar Vishwakarma and Ozgur Kisi.
