# Supplementary material for: Comparative evaluation of hybrid and individual models for predicting soybean yellow mosaic virus incidence
Source: Sci Rep. 2025 May 6;15:15790. doi: 10.1038/s41598-025-99427-5 (PMC12056175; doi:10.1038/s41598-025-99427-5)
Supplement: Supplementary file 1 — Supplementary Material 1 [file 41598_2025_99427_MOESM1_ESM.docx]

**Supplementary Information**

**Table S1.** Details of error percentage by SMLR and PCA-SMLR model.

| **Model** | **Year** | **AUDPC Observed** | **AUDPC Predicted** | **Error (%)** |
| --- | --- | --- | --- | --- |
| SMLR | 2017 | 85.15 | 233.81 | 63.58 |
|  | 2018 | 219.90 | 230.44 | 4.57 |
|  | 2019 | 376.42 | 200.83 | -87.43 |
|  | 2020 | 323.33 | 256.65 | -25.98 |
| PCA-SMLR | 2017 | 85.15 | 201.61 | 57.76 |
|  | 2018 | 219.90 | 194.94 | -12.81 |
|  | 2019 | 376.42 | 232.31 | -62.04 |
|  | 2020 | 323.33 | 204.29 | -58.27 |

**Table S2**. Details of error percentage by ANN and PCA-ANN model.

| **Model** | **Year** | **AUDPC Observed** | **AUDPC Predicted** | **Error (%)** |
| --- | --- | --- | --- | --- |
| ANN | 2017 | 85.15 | 81.99 | 3.72 |
|  | 2018 | 219.90 | 210.63 | 4.22 |
|  | 2019 | 376.42 | 343.63 | 8.71 |
|  | 2020 | 323.33 | 322.24 | 0.33 |
| PCA-ANN | 2017 | 85.15 | 91.89 | -7.91 |
|  | 2018 | 219.90 | 220.33 | -0.19 |
|  | 2019 | 376.42 | 362.17 | 3.79 |
|  | 2020 | 323.33 | 313.81 | 2.94 |

**Table S3.** Details of error percentage by LASSO and PCA-LASSO model.

| **Model** | **Year** | **AUDPC Observed** | **AUDPC Predicted** | **Error (%)** |
| --- | --- | --- | --- | --- |
| LASSO | 2017 | 85.15 | 199.51 | -134.30 |
|  | 2018 | 219.90 | 198.21 | 9.87 |
|  | 2019 | 376.42 | 225.60 | 40.07 |
|  | 2020 | 323.33 | 217.43 | 32.75 |
| PCA-LASSO | 2017 | 85.15 | 208.52 | -144.87 |
|  | 2018 | 219.90 | 192.13 | 12.63 |
|  | 2019 | 376.42 | 161.91 | 56.98 |
|  | 2020 | 323.33 | 116.47 | 63.98 |

**Table S4.** Details of error percentage by RR and PCA-RR model.

| **Model** | **Year** | **AUDPC Observed** | **AUDPC Predicted** | **Error (%)** |
| --- | --- | --- | --- | --- |
| RR | 2017 | 85.15 | 214.89 | -152.35 |
|  | 2018 | 219.90 | 219.58 | 0.15 |
|  | 2019 | 376.42 | 228.48 | 39.30 |
|  | 2020 | 323.33 | 232.00 | 28.24 |
| PCA-RR | 2017 | 85.15 | 210.71 | -147.44 |
|  | 2018 | 219.90 | 233.58 | -6.22 |
|  | 2019 | 376.42 | 220.75 | 41.35 |
|  | 2020 | 323.33 | 209.11 | 35.32 |

**Table S5.** Details of error percentage by ELNET and PCA-ELNET model.

| **Model** | **Year** | **AUDPC Observed** | **AUDPC Predicted** | **Error (%)** |
| --- | --- | --- | --- | --- |
| ELNET | 2017 | 85.15 | 214.89 | -152.35 |
|  | 2018 | 219.90 | 219.58 | 0.15 |
|  | 2019 | 376.42 | 228.48 | 39.30 |
|  | 2020 | 323.33 | 232.00 | 28.24 |
| PCA-ELNET | 2017 | 85.15 | 266.71 | -213.20 |
|  | 2018 | 219.90 | 291.29 | -32.46 |
|  | 2019 | 376.42 | 257.94 | 31.47 |
|  | 2020 | 323.33 | 261.53 | 19.11 |

**Table S6.** Details of error percentage by SMLR-ANN and PCA-SMLR-ANN model.

| **Model** | **Year** | **AUDPC Observed** | **AUDPC Predicted** | **Error (%)** |
| --- | --- | --- | --- | --- |
| SMLR-ANN | 2017 | 233.81 | 231.47 | 1.00 |
|  | 2018 | 230.44 | 237.62 | -3.12 |
|  | 2019 | 200.83 | 206.98 | -3.06 |
|  | 2020 | 256.65 | 259.73 | -1.20 |
| PCA-SMLR-ANN | 2017 | 201.61 | 200.88 | 0.36 |
|  | 2018 | 194.94 | 193.37 | 0.80 |
|  | 2019 | 232.31 | 232.73 | -0.18 |
|  | 2020 | 204.29 | 201.64 | 1.29 |

**
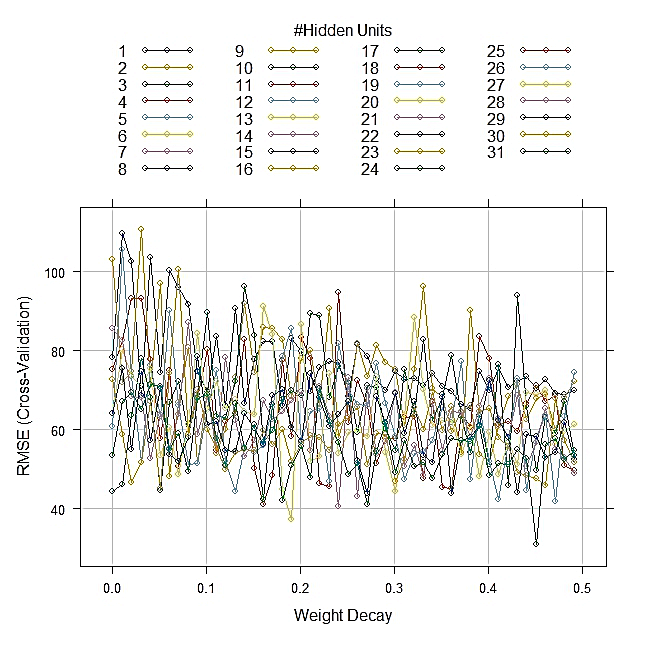
**

**Fig. S1.** Size (No. of nodes in the hidden layer) and decay values utilized by ANN.

**
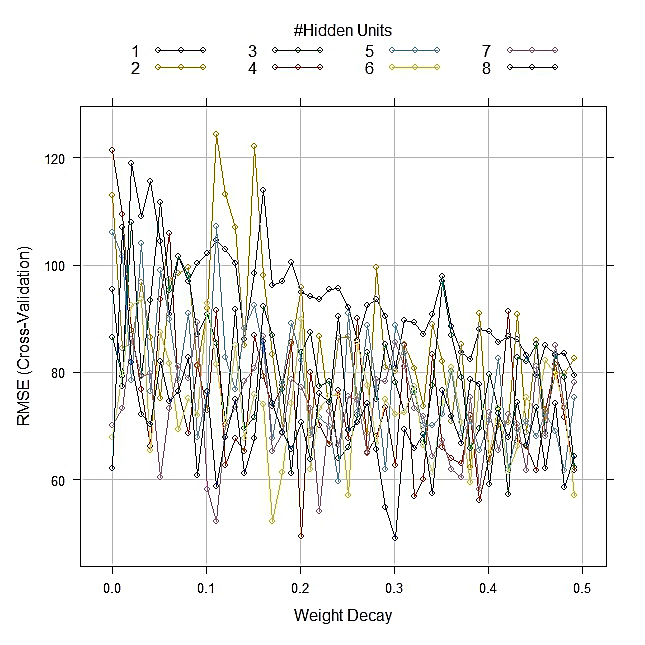
**

**Fig. S2.** Size (No. of nodes in the hidden layer) and decay values utilized by PCA-ANN.

**
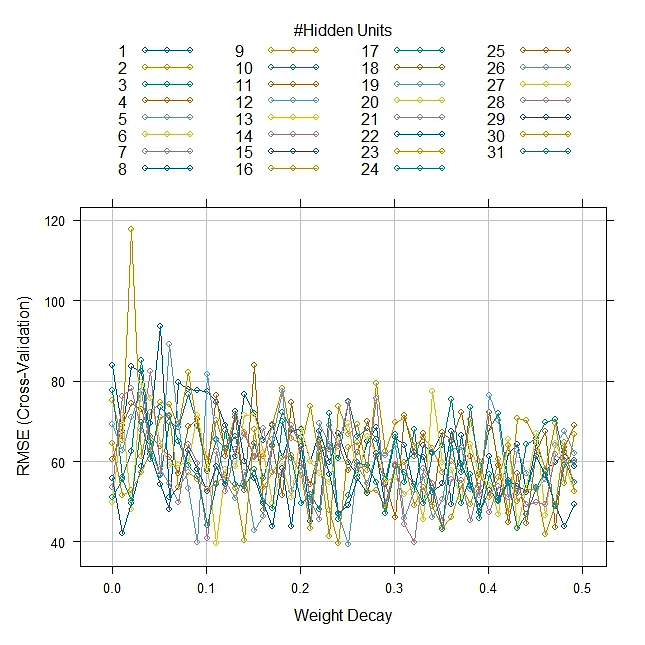
**

**Fig. S3.** Size (No. of the hidden layers) and decay values utilized by SMLR-ANN.

**
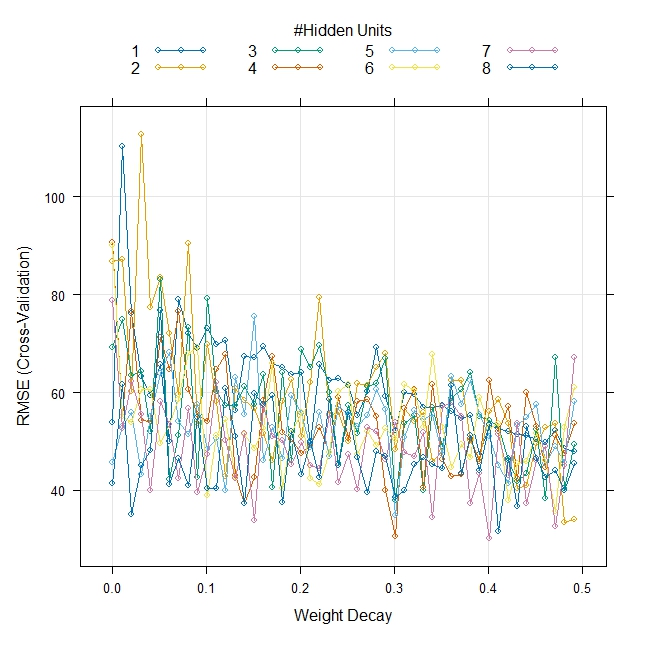
**

**Fig. S4.** Size (No. of hidden layers) and decay values utilized by PCA-SMLR-ANN.
